# Current Insights Into Adrenal Insufficiency in the Newborn and Young Infant

**DOI:** 10.3389/fped.2020.619041

**Published:** 2020-12-14

**Authors:** Federica Buonocore, Sinead M. McGlacken-Byrne, Ignacio del Valle, John C. Achermann

**Affiliations:** Genetics & Genomic Medicine Research and Teaching Department, UCL Great Ormond Street Institute of Child Health, London, United Kingdom

**Keywords:** adrenal insufficiency, Addison's disease, adrenal hypoplasia, congenital adrenal hyperplasia, glucocorticoid, DAX-1, MIRAGE syndrome, genetic testing

## Abstract

Adrenal insufficiency (AI) is a potentially life-threatening condition that can be difficult to diagnose, especially if it is not considered as a potential cause of a child's clinical presentation or unexpected deterioration. Children who present with AI in early life can have signs of glucocorticoid deficiency (hyperpigmentation, hypoglycemia, prolonged jaundice, poor weight gain), mineralocorticoid deficiency (hypotension, salt loss, collapse), adrenal androgen excess (atypical genitalia), or associated features linked to a specific underlying condition. Here, we provide an overview of causes of childhood AI, with a focus on genetic conditions that present in the first few months of life. Reaching a specific diagnosis can have lifelong implications for focusing management in an individual, and for counseling the family about inheritance and the risk of recurrence.

## Introduction

Adrenal insufficiency (AI) is a potentially life-threatening condition that needs urgent diagnosis and treatment (1–4). AI is relatively rare in early life, affecting approximately 1:5,000–10,000 children, and its features can be non-specific. Children can be initially mis-diagnosed as having sepsis, metabolic conditions, or cardiovascular disease, highlighting the need to consider adrenal dysfunction as a differential diagnosis for an unwell or deteriorating infant. Prompt recognition allows the correct investigations to be undertaken urgently and definitive management to be established.

AI can be broadly divided into *secondary* causes, due to disruption of hypothalamic or pituitary (corticotrope) ACTH release, and *primary* causes, which affect the adrenal gland itself. Although some conditions have fairly typical presentation patterns and ages of onset, there is often a spectrum of features, and milder variants may produce partial or delayed onset forms of classic conditions (5, 6). Associated features can sometimes give a clue to the diagnosis.

Here, we provide a brief summary of the genetic causes of AI that tend to present in the neonatal period or first few months of life, and the implications of making a specific genetic diagnosis for management. While the focus of this minireview is very much on genetic causes, physical causes (such as adrenal hemorrhage or infiltration) should not be overlooked.

## Secondary Adrenal Insufficiency

**Figure 1:**
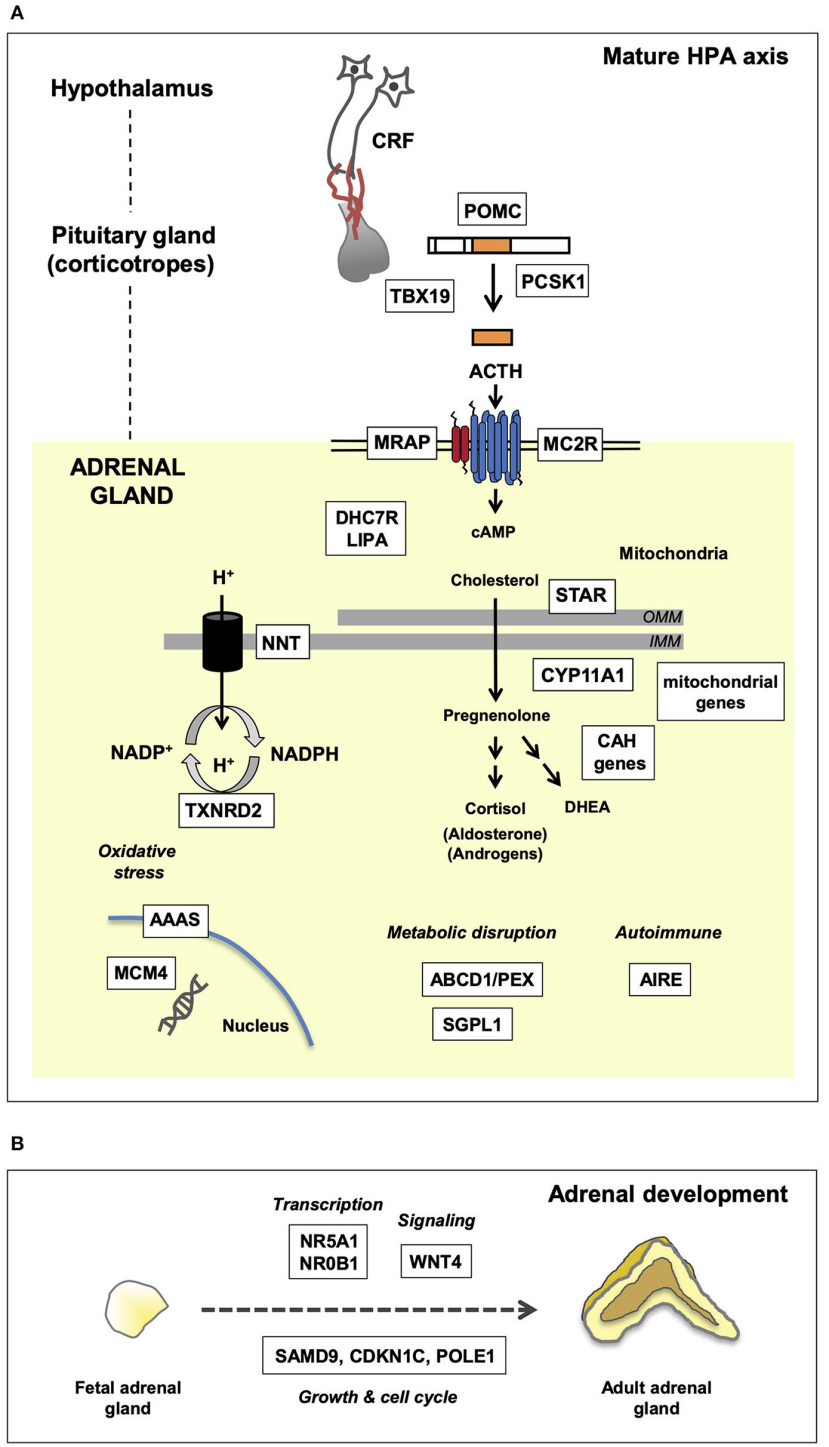
Genetic mechanisms of pediatric adrenal insufficiency (AI) along the hypothalamo-pituitary-adrenal (HPA) axis. Key genes are shown in white boxes. **(A)** Genetic causes of AI as they relate to the mature HPA axis. These genes are required for a multitude of key enzymatic and biochemical processes occurring within the nucleus, mitochondria, and cytoplasm. Disruption of these genes gives rise to the clinical phenotypes discussed in the text. OMM, outer mitochondrial membrane; IMM, inner mitochondrial membrane. **(B)** Overview of adrenal development and key genes associated with adrenal hypoplasia. Adrenal hypoplasia is mediated by disruption of key genes required for normal fetal adrenal development. These genes are involved in transcription, signaling, and growth/cell cycle processes.

Secondary AI is caused by impaired ACTH synthesis and release from pituitary corticotrope cells (Figure 1A). ACTH deficiency can be isolated or can occur as part of a combined (multiple) pituitary hormone deficiency (CPHD) due to defects in hypothalamo-pituitary function (Table 1). Usually glucocorticoid release is affected, whereas disturbances in mineralocorticoid function and salt balance are unusual as aldosterone synthesis is primarily under the control of the renin-angiotensin system.

**Table 1:** Selected monogenic causes of adrenal insufficiency in children.

**Condition**	**Gene (PROTEIN if different)**	**Inheritance**	**Associated features**
**Secondary adrenal insufficiency**			
CPHD	*GLI1, HESX1, LHX3, LHX4, SOX3, SOX2, OTX2* and others; also *PROP1, GH1* (delayed)	Variable	Many variable associated features including holoprosencephaly, tooth abnormalities (*GLI1*); septo-optic dysplasia (esp. *HESX1*); short rigid neck, hearing loss (*LHX3*); anophthalmia (*SOX2, OTX2*)
Isolated ACTH deficiency	*TBX19* (TPIT)	AR	
POMC deficiency	*POMC* (Pro-opio-melanocortin)	AR	Obesity, red hair
Prohormone convertase deficiency	*PCSK1* (PC-1)	AR	Obesity, hypoglycemia, hypogonadotropic hypogonadism
**Primary adrenal insufficiency**			
***Disorders of steroidogenesis***			
Smith–Lemli–Opitz syndrome	*DHC7R* (7-dehydrocholesterol reductase)	AR	Syndactyly, polydactyly, facial features, microcephaly, cardiac defects, gastrointestinal features, hypospadias/undescended testes
Congenital lipoid adrenal hyperplasia^*a*^	*STAR*	AR	46,XY DSD, impaired gonadal steroidogenesis
P450 side chain cleavage def.^*a*^	*CYP11A1* (P450scc)	AR	46,XY DSD, impaired gonadal steroidogenesis
21-hydroxylase def. (CAH)	*CYP21A2* (P450c21)	AR	46,XX DSD, virilization, early puberty
11β-hydroxylase def. (CAH)	*CYP11B1* (P450c11)	AR	46,XX DSD, virilization, early puberty, hypertension
3β-hydroxysteroid dehydrogenase type 2 def. (CAH)	*HSD3B2* (3β-HSD2)	AR	46,XY DSD, impaired gonadal steroidogenesis; 46,XX DSD, clitoromegaly
17α-hydroxylase/17,20-lyase def. (CAH)	*CYP17A1* (P450c17)	AR	46,XY DSD, impaired gonadal steroidogenesis, hypertension
P450 oxidoreductase def. (CAH)	*POR* (P450 oxidoreductase)	AR	Antley-Bixler syndrome (craniosynostosis, skeletal features, choanal atresia), atypical genitalia (46,XY and 46,XX), impaired gonadal steroidogenesis at puberty
***Adrenal hypoplasia***			
X-linked AHC	*NR0B1* (DAX-1)	X-linked	Hypogonadotropic hypogonadism, impaired spermatogenesis
Steroidogenic factor-1	*NR5A1* (SF-1)	AD, AR, SLD	46,XY DSD, asplenia
IMAGe syndrome	*CDKN1C*	Imprinted	IUGR, metaphyseal dysplasia, genital anomalies
IMAGe-like syndrome with immunodeficiency	*POLE1*	AR	IUGR, skeletal changes, adrenal hypoplasia, genital anomalies, infections/immunodeficiency, developmental dysplasia of the hip, post-natal growth restriction/facial features
MIRAGE syndrome	*SAMD9*	AD (*de novo*)	Infections, IUGR/preterm, gonadal dysfunction, enteropathy, anemia, thrombocytopenia; risk of monosomy 7 and myelodysplastic syndrome
SERKAL syndrome	*WNT4*	AR	46,XX DSD, renal dysgenesis, pulmonary hypoplasia
***ACTH-resistance and related conditions***			
FGD1	*MC2R* (ACTH receptor)	AR	Tall stature (pre-treatment)
FGD2	*MRAP* (MC2R-accessory protein)	AR	
Nicotinamide nucleotide transhydrogenase	*NNT*	AR	Early puberty
Thioredoxin reductase 2	*TXNRD2*	AR	Heart defects
Triple A syndrome (Allgrove syndrome)	*AAAS* (Aladin)	AR	Achalasia, alacrima, ataxia/neurological involvement, hyperkeratosis
Minichromosome maintenance-4	*MCM4*	AR	Natural killer cell defects, microcephaly, post-natal growth failure
***Metabolic conditions***			
Sphingosine-1-phosphate lyase 1 insufficiency	*SGPL1*	AR	Steroid-resistant nephrotic syndrome, ichthyosis, neurological involvement, hypothyroidism, cryptorchidism
X-linked adrenoleukodystrophy	*ABCD1*	X-linked	Neurological dysfunction
Zellweger spectrum disorders (incl. neonatal adrenoleukodystrophy)	*PEX* genes; related genes (Peroxins)	AR	Neurological, facial features, hepatic dysfunction
Mitochondrial disorders (Kearne-Sayre syndrome; Pearson syndrome; others)	Mitochondrial DNA, *MRPS7, NDUFAF5, GFER*	Maternal or AR	Variable multisystem features
Wolman disease	*LIPA* (Cholesterol ester)	AR	Failure to thrive, hepatosplenomegaly, adrenal calcification
***Autoimmune conditions***			
APS1 (APECED)	*AIRE* (Autoimmune regulator)	AD, AR	Hypoparathyroidism, mucocutaneous candidiasis, alopecia, pernicious anemia, other autoimmune features

CPHD, combined (multiple) pituitary hormone deficiency; AD, autosomal dominant; AR, autosomal recessive; CAH, congenital adrenal hyperplasia, DSD, differences (disorders) in sex development; SLD, sex-limited dominant; IUGR, intrauterine growth restriction; FGD, familial glucocorticoid deficiency.

a Partial defects in STAR and P450scc can present with predominant glucocorticoid insufficiency in childhood and mimic FGD.

*Modified with permission from Lin and Achermann (7). Copyright 2004 Blackwell Publishing Ltd*.

### Combined Pituitary Hormone Deficiency

Several genetic causes of CPHD are reported (e.g., *GLI1, HESX1, LHX3, LHX4, SOX3, SOX2* and others) (8). Pituitary ACTH insufficiency usually occurs together with loss of other anterior pituitary hormones (GH, TSH, LH/FSH). Concomitant GH and ACTH insufficiency often causes hypoglycemia in young children, and a small penis and undescended testes may be a sign of congenital gonadotropin insufficiency in boys (9). Other associated features include septo-optic dysplasia or specific associations such as micro-ophthalmia (*SOX2, OTX2*). Disruption of *PROP1* or *GH1* can cause ACTH insufficiency in later life (10).

### Isolated ACTH Deficiency

Isolated ACTH deficiency can occur due to disruption of TPIT (*TBX19*), or with associated features due to defects in pro-opiomelanocortin (*POMC*) or pro-hormone convertase-1 (PC-1/*PCSK1*).

TPIT is a transcription factor that regulates synthesis of POMC in pituitary corticotrope cells, but not in other POMC producing cells of the body (e.g., skin, hypothalamus) (11). POMC is a precursor molecule that is cleaved to release ACTH along with other peptides (e.g., alpha-MSH, beta-endorphin) (Figure 1A). Children with severe disruption of TPIT usually present with evidence of glucocorticoid insufficiency, such as hypoglycemia or hypoglycemic seizures, and prolonged conjugated hyperbilirubinemia in the first few weeks of life (12, 13). This contrasts to *late-onset* isolated ACTH insufficiency, where the molecular basis is currently unknown.

Defects in POMC itself also result in ACTH insufficiency and adrenal dysfunction in early infancy (14). Children have red (or auburn) hair and pale skin due to MSH deficiency, and profound hyperphagia and weight gain from later infancy due to hypothalamic POMC disruption (15). MC4R agonists, which mimic MSH, have had promising results in suppressing hyperphagia in this condition, so it is an important diagnosis to make (16).

Disruption of the cleavage enzyme prohormone convertase-1 (PC-1, *PCSK1*) also presents with ACTH insufficiency, together with hypoglycemia, malabsorptive diarrhea, obesity, and hypogonadism (17, 18). This diagnosis is rare.

## Primary Adrenal Insufficiency

An overview of monogenic causes of primary adrenal insufficiency (PAI) in childhood is shown in Table 1 and Figures 1A,B, together with inheritance patterns and associated features. Here, we focus primarily on key genetic causes of PAI that present in the first few months of life. Disorders of salt-balance (e.g., aldosterone synthase deficiency) are not included.

### Disorders of Steroidogenesis

#### Smith–Lemli–Opitz Syndrome

Smith–Lemli–Opitz syndrome is a defect in cholesterol biosynthesis due to disruption of the enzyme 7-dehydrocholesterol reductase (*DHCR7*) (19). Common findings in infancy are microcephaly, cleft palate, syndactyly of the second and third toes, post-axial polydactyly, congenital heart defects, gastrointestinal issues (e.g., pyloric stenosis), atypical genital and undescended testes (46,XY) and characteristic facial features (20). AI or impaired stress response can occur, but are surprisingly rare (21, 22). Elevated 7-dehydrocholesterol is diagnostic, coupled with genetic testing.

#### Early Steroidogenic Defects (*STAR/CYP11A1*)

Steroidogenic acute regulatory protein (*STAR*) plays a key role in cholesterol transport into the mitochondria, whereas the P450 cholesterol side-change cleave enzyme (P450scc, encoded by *CYP11A1*) catalyzes the conversion of cholesterol to pregnenolone (Figure 1) (23, 24). These proteins are both required for adrenal (glucocorticoid, mineralocorticoid) and gonadal (testosterone, estrogen) steroid synthesis. Severe disruption causes salt-losing AI in all children and female-typical external genitalia in 46,XY infants. The onset of PAI usually occurs at around 3–4 weeks of age in complete STAR deficiency (also known as “congenital lipoid adrenal hyperplasia”), as build-up of intracellular cholesterol takes time to cause cellular damage (“two-hit hypothesis”) (25). In contrast, infants with a severe P450scc deficiency usually present with salt-losing PAI at around 7-10 days (26, 27). Partial disruption of these proteins results in predominant glucocorticoid insufficiency in childhood (27–31).

#### Congenital Adrenal Hyperplasia

The most common cause of PAI in the first month of life is congenital adrenal hyperplasia (CAH) (23). In virtually all populations, 21-hydroxylase deficiency (21-OHD, *CYP21A2*) is most prevalent, with an incidence of 1:10,000–1:20,000 (although geographical hotspots occur) (32, 33). Other rare forms of CAH include 11 beta-hydroxylase deficiency (*CYP11B1*) (especially in Jewish populations originating from Morocco), 3 beta-hydroxysteroid dehydrogenase deficiency (*HSD3B2*), 17 alpha-hydroxylase deficiency (*CYP17A1*) and P450 oxidoreductase deficiency (*POR)*, all of which have specific presentations and biochemical profiles (Table 1) (23).

Approximately 80% of 46,XX girls with confirmed 21-OHD have atypical genitalia at birth, so any baby with genital differences and non-palpable gonads should be considered as having 21-OHD until proven otherwise (33, 34). Progressive salt loss usually results in hyperkalemia and hyponatremia at around 5–7 days of life, mandating urgent monitoring and treatment once the diagnosis is made. Boys (46,XY) with 21-OHD have no obvious signs at birth and usually present in a salt-losing adrenal crisis between 1 and 2 weeks of age, so many countries include 17-hydroxyprogesterone in their newborn screening program. More detailed reviews of CAH and its management are presented elsewhere (32, 33).

### Adrenal Hypoplasia

Adrenal hypoplasia is an underdevelopment of the adrenal glands, which often presents with PAI in early life (Figure 1B). Usually this is an X-linked condition, or sometimes associated with intrauterine growth restriction (IUGR) (fetal growth restriction, FGR) syndromes.

#### X-Linked Adrenal Hypoplasia

X-linked congenital adrenal hypoplasia (adrenal hypoplasia congenita, AHC) primarily affects boys and is associated with disruption of the nuclear receptor, DAX-1 (encoded by *NR0B1*) (35, 36). This condition presents with salt-losing PAI in the first 2 months of life (40%), or more insidiously with AI in childhood (37). Late-onset forms of the condition have also been described (38–40).

X-linked AHC has three main features: PAI, hypogonadotropic hypogonadism (HH) in adolescence, and impaired spermatogenesis (41). Some boys may paradoxically have *macro*phallia at birth, and initial presentation with either isolated mineralocorticoid insufficiency or isolated glucocorticoid insufficiency is reported (42, 43). Growth hormone insufficiency has also been diagnosed in a small subset of boys (37, 44–46).

Boys with PAI and HH in adolescence almost invariably have X-linked AHC, especially if there is a family history of X-linked adrenal dysfunction. Even without such a history, we found that approximately 40% of boys presenting with salt-losing AI in the first two months of life had X-linked AHC, once more common conditions such as CAH had been excluded (47). Approximately two-thirds of boys have pathogenic missense or loss-of-function (stop gain, frameshift) variants in DAX-1/*NR0B1*, around one-sixth have a localized deletion of this gene on the X-chromosome (Xp21), and one-sixth have a larger Xp contiguous gene deletion syndrome that can involve genes causing glycerol kinase deficiency (*GKD*), ornithine transcarbamylase deficiency (*OTC*) and Duchenne Muscular Dystrophy (*DMD*) (47). Very rarely, girls have X-linked AHC due to skewed X-inactivation (48). Establishing this diagnosis early allows prompt recognition and management of both the PAI and potential associated conditions (39). Families can be counseled about risk in brothers or in the maternal family, and presymptomatic boys diagnosed (49).

#### Steroidogenic Factor-1 (SF-1/*N5A1*)

Steroidogenic factor-1 (SF-1/*NR5A1*) is a related nuclear receptor considered as a “master-regulator” of adrenal and reproductive development (36). Severe disruption of SF-1 has very rarely been associated with early-onset PAI in 46,XX girls and 46,XY phenotypic female babies with testicular dysgenesis, usually due to disruption of key DNA-binding elements of this transcription factor (50, 51). In contrast, more than 200 individuals and families with heterozygous pathogenic variants in SF-1/*NR5A1* have been reported, having a wide spectrum of reproductive phenotypes (from gonadal dysgenesis through to male factor infertility or primary ovarian insufficiency and ovotesticular DSD) (36, 52–54). To date, adrenal function is normal in most of these individuals.

#### IMAGe Syndrome (*CDKN1C* and *POLE1*)

AI associated with IUGR/FGR can occur as part of IMAGe syndrome (intrauterine growth restriction, metaphyseal dysplasia, adrenal hypoplasia, genitourinary anomalies) (55, 56). Children usually present with salt-losing PAI in early life. Other variable features include frontal bossing, impaired glucose tolerance, and hearing loss.

Classic IMAGe syndrome is associated with gain-of-function variants in the cell-cycle repressor, CDKN1C (56). This is a paternally-imprinted (maternally-expressed gene), so is usually inherited from the mother, but can occur *de novo*. IMAGe-associated pathogenic variants are localized within a very specific motif in the PCNA-binding motif of CDKN1C, causing impaired cell cycle S-phase progression (57). Variants neighboring this motif can cause IUGR/Russell-Silver Syndrome phenotypes with normal adrenal function (58, 59). Of note, loss-of-function of CDKN1C is associated with Beckwith-Wiedemann syndrome, an “overgrowth” syndrome, highlighting how different changes in one gene can have opposing phenotypes (58).

Recently, an “IMAGe-like” syndrome with AI and immunodeficiency (infections, lymphopenia, hypogammaglobulinemia) has been reported (60). These children have profound postnatal growth restriction, distinctive facial features, hip dysplasia and hypoplastic patellae. This condition results from pathogenic biallelic variants in polymerase epsilon-1 (*POLE1*, Pol ε), often involving a heterozygous intronic variant (c.1686 + 32C>G). POLE1 is a DNA polymerase that interacts with PCNA in S-phase DNA replication.

#### MIRAGE Syndrome (*SAMD9*)

Another multisystem growth restriction syndrome associated with adrenal hypoplasia is MIRAGE syndrome (myelodysplasia, infections, restriction of growth, adrenal hypoplasia, genital phenotypes, enteropathy) (61, 62). Infants with severe forms of MIRAGE are born preterm and develop salt-losing PAI in early life. Recurrent infections (viral, bacterial and fungal), anemia/thrombocytopenia, nephropathy, severe enteropathy, esophageal reflux and aspiration are common, and 46,XY children have penoscrotal hypospadias or gonadal (testicular) dysfunction with a female phenotype. Mortality is high and children who survive show long-term growth restriction. As many of these features are found in sick, preterm, growth-restricted babies, it is likely that this condition is under-diagnosed.

MIRAGE syndrome results from heterozygous gain-of-function missense mutations in the growth repressor, sterile alpha domain containing 9 (*SAMD9*) (61, 62). These changes usually occur *de novo* and restrict cell growth and division, potentially through reduced recycling of growth factor receptors. SAMD9 also plays a role in innate viral immunity and host defense.

One interesting aspect of MIRAGE syndrome is how secondary genetic events can dynamically modify the phenotype through “revertant mosaicism.” For example, development of progressive, somatic monosomy 7 in *cis* (i.e., on the same allele) “removes” the deleterious gain-of-function mutation in SAMD9 allowing a clonal growth advantage of these affected cells, especially in the hematopoietic system, and reversal of the postnatal anemia and thrombocytopenia (61, 62). However, monosomy 7 is linked to the development of myelodysplastic syndrome, which can lead to leukemia if other genetic changes occur. Interestingly, somatic *loss-of-function* (nonsense or frameshift) changes or uniparental disomy in *cis* can also “remove” the mutant allele and ameliorate the phenotype (5, 62–64). Increasingly, children with milder MIRAGE-like features are being reported, many with normal adrenal function (65).

#### SeRKAL Syndrome (*WNT4*) and Other Associations

SeRKAL syndrome (female sex reversal and dysgenesis of kidneys, adrenals, and lungs) has been reported in a single family with homozygous disruptive mutations in WNT4, a signaling molecule implicated in adrenal development (66). Other historic reports have rarely described AI with Pena–Shokeir syndrome type I (*DOK7, RAPSN*), pseudotrisomy 13, Galloway–Mowat syndrome (*WDR73*), Pallister–Hall syndrome (*GLI3*, with pituitary defects) and Meckel–Gruber syndrome (*MKS1*) (67).

### ACTH Resistance-Like Conditions

Another important group of conditions causing PAI in childhood are ACTH-resistance conditions (also known as Familial Glucocorticoid Deficiency, FGD) and related disorders (68). Some of these may present in early infancy.

#### Familial Glucocorticoid Deficiency Type 1 (*MC2R*)

Familial Glucocorticoid Deficiency Type 1 (FGD1) is a recessive condition that results from pathogenic variants in the ACTH receptor (melanocortin 2 receptor, *MC2R*) (68, 69). Children sometimes present in the first weeks of life with signs of cortisol insufficiency (hypoglycemia/convulsions, prolonged jaundice) and marked hyperpigmentation. Genuine mineralocorticoid insufficiency is very rare, but transient salt loss or dilutional hyponatremia can occur, sometimes leading to a misdiagnosis of adrenal hypoplasia (70, 71). FGD1 can also present later in childhood with recurrent infections, hyperpigmentation, and lethargy. Generally, children respond very well to glucocorticoid replacement, but ACTH concentrations can be difficult to suppress.

#### Familial Glucocorticoid Deficiency Type 2 (*MRAP*)

A similar form of ACTH-resistance results from disruption of the melanocortin 2 receptor accessory protein, MRAP (72). MRAP traffics MC2R to the adrenal cell membrane surface, so disruption of its function (usually due to splicing defects in exon 3) impairs ACTH signaling (68, 73, 74). Affected children usually present with severe glucocorticoid insufficiency and hyperpigmentation in the first few months of life.

#### Disorders Associated With Oxidative Stress (*NNT, TNXRD2*)

Defects in nicotinamide nucleotide transhydrogenase (*NNT*) are a well-established cause of isolated PAI in children, and occasionally additional features such as early puberty have been reported (68, 75, 76). This condition mostly presents after 1 year of age but has been reported as early as 4 months of age. To date, defects in thioredoxin reductase 2 (*TNXRD2*) are reported in a single family (sometimes with cardiac defects), and present in mid- or later childhood (77).

#### Triple A Syndrome (Allgrove Syndrome)

Triple A syndrome is a well-established condition linking PAI (“Addison disease”), with alacrima and achalasia of the esophagus (78, 79). This condition results from disruption of the protein aladin (encoded by *AAAS*), a potential nucleoporin component that may also influence cellular stress (80–82). Alacrima is often present from birth but is difficult to diagnose. Other features usually develop in childhood, or in the second decade of life (83, 84). Progressive neurological and autonomic dysfunction can also co-occur, so this is an important diagnosis to consider.

#### Other Related Forms of PAI

Disruption of minichromosome maintenance 4 (*MCM4*) causes mild PAI together with short stature and immunodeficiency (85). To date, this is only reported in individuals of Irish Traveller ancestry, and typically manifests in mid-childhood. As noted above, partial (non-classic) high steroidogenic blocks (*STAR* and *CYP11A1*) can present in childhood with PAI. Non-classic STAR defects are sometimes termed FGD3 (27–31).

### Metabolic Conditions

Several metabolic conditions are associated with PAI but the presentation and features can be variable (86).

#### Sphingosine-1-Phosphate Lyase (*SGPL1*) Deficiency

SGPL1 deficiency is a novel sphingolipidosis that results from impaired breakdown of sphingosine 1-phosphate (87–89). Key features are PAI (sometimes with adrenal calcifications) and steroid-resistant nephrotic syndrome (SRNS), as well as ichthyosis, neurological dysfunction, primary hypothyroidism, lymphopenia and undescended testes. Many children present with PAI in the first year of life (hyperpigmentation, or adrenal crisis), although some first present with SRNS and the use of steroid treatment may delay the adrenal phenotype. Other features are variable and can appear or progress with time.

#### Adrenoleukodystrophy and Related-Conditions

Adrenoleukodystrophy (ALD) is a very important cause of PAI because of associated progressive neurological features (90). The X-linked form of ALD due to defects in *ABCD1* usually presents in childhood, and sometimes with adrenal-only features. Thus, all boys with undiagnosed causes of PAI should have long-chain fatty acids measured and there are some calls for newborn screening to increase early detection, since allogenic hematopoietic stem cell transplantation may reduce the progression of cerebral X-ALD in patients with early stages of disease, and hematopoietic stem cell gene therapy has been investigated (91–94). In contrast, “neonatal adrenal leukodystrophy” is now classified as part of the “Zellweger Spectrum Disorders” (with Zellweger syndrome/cerebrohepatorenal syndrome, infantile Refsum disease and rhizomelic chondrodysplasia punctata type 1) (95). This spectrum of disorders results from defects in peroxisomal function (13 different *PEX* genes and others) and has many features including hypotonia, seizures, hepatic dysfunction and renal cysts. PAI has been reported, usually in childhood or with an impaired stress response (96), so screening after 1 year of age has been recommended (95).

#### Mitochondrial Disorders

Mitochondrial defects have a range of causes and presenting features. Adrenal dysfunction occurs in rare cases, more often associated with large scale mitochondrial DNA deletions (e.g., Kearns-Sayre and Pearson syndromes), but also pathogenic variants in other related genes (e.g., *MK-TK, MRPS7, QRSL1, NDUFAF5, GFER*) (86, 97).

#### Wolman Disease

Wolman disease (primary xanthomatosis) results from disruption of lysosomal acid lipase (*LIPA*), and is associated with AI (often with adrenal calcifications), failure to thrive, hepatosplenomegaly and anemia in the first few months of life. It is a lysosomal storage disorder that is usually fatal, although improvements with enzyme replacement treatment (sebelipase alfa) are reported (98–100).

### Autoimmune Conditions

Although autoimmune “Addison disease” is the most common cause of AI in adolescents and adults, autoimmune PAI is rare in children (101). The best-established condition is Autoimmune Polyglandular Syndrome type 1 (APS1, also known as APECED), due to defects in autoimmune regulator (*AIRE*). Early features can include mucocutaneous candidiasis and rarely hypoparathyroidism (hypocalcemia). PAI and other associations usually occur in childhood or later life.

### Other Causes of PAI

Physical causes of AI such as hemorrhage or infiltration (e.g., neuroblastoma) should not be overlooked. Unilateral adrenal hemorrhages detected by imaging are common (1:200–500 newborn), but usually asymptomatic (102). Symptomatic bilateral hemorrhages are rare but can cause profound AI. As in older children, prolonged administration of glucocorticoids for other conditions can suppress the hypothalamo-pituitary adrenal (HPA) axis and cause AI if withdrawal is rapid.

Transient, relative AI has been described in some very preterm babies, or in sick newborn children under stress. The physiological basis of this is unclear, but steroid supplements have been used in some situations (103).

## Importance of Making a Specific Diagnosis

Making a specific genetic diagnosis has several benefits. It allows tailored treatment of the specific underlying hormonal defect (such as the need for ongoing mineralocorticoid replacement or not) and permits the surveillance, early recognition, and prompt treatment of associated extra-adrenal features (16, 61, 62, 70, 71, 87, 98).

Reaching a specific genetic diagnosis also has wider implications for the family, especially as these conditions have a range of inheritance patterns (e.g., autosomal recessive, dominant/*de novo*; X-linked, imprinted). This information guides genetic counseling during future pregnancies, and potentially allows pre-symptomatic diagnosis and treatment in relatives with subclinical disease (49).

## Genetic Testing for PAI in Early Life

Traditionally, genetic testing has relied on Sanger sequencing of *candidate genes* one at a time. This approach may still have a role in common conditions such as 21-OHD (*CYP21A2*), where there is a specific biochemical profile, well-established pathogenic variants, and a pseudogene that can complicate analysis, or in X-linked AHC (DAX-1/*NR0B1*) when well-established associations (e.g., HH) or inheritance patterns (e.g., X-linked) are present.

However, associated features or pathognomonic biochemical patterns are often not present when an infant presents with PAI, so “next generation” sequencing (NGS) approaches are increasingly time- and cost-effective.

Access to services varies from country to country, but *targeted “panels”* to analyze all key PAI genes at once are increasingly available as a clinical service. In addition, several studies have shown how “trio” *whole exome sequencing* (WES) can help diagnose sick infants and children, especially when there are complex, multisystem features, and exome analysis has been reported to help in the diagnosis of children with PAI (104). *Whole genome sequencing* (WGS) will become increasingly available and has potential advantages and disadvantages at present compared to panels/WES.

In general, genetic testing for PAI has a high diagnosis rate, certainly when compared to other pediatric endocrine conditions such as congenital hypothyroidism and hypothalamo-pituitary hormone deficiencies. For example, in a national cohort study of rare, undiagnosed PAI in Turkey (with CAH and obvious metabolic causes excluded), a specific genetic diagnosis was reached in 80–90% of children (105), although the diagnostic yield of autosomal recessive conditions was high due to high consanguinity rates. New genetic causes may still emerge as our understanding of human adrenal development expands (106).

Finally, founder effects and genetic “hotspots” can be very important in identifying a specific genetic cause of PAI and taking a history of family ancestry is key. For example, in Turkey the *MRAP* splice variant cIVS3ds + 1delG is found in the West, whereas P450scc/CYP11A1 p.R451W occurs in Eastern regions (105). As families migrate around the world, founder effects are seen in children born in other countries. Knowing a specific hotspot can allow focused and cost-effective screening of “at risk” family members before the onset of PAI.

## Conclusions

AI is an important diagnosis to consider in any sick newborn infant and prompt investigation and treatment is essential. Genetic testing is increasingly useful for finding a specific cause, predicting associated features, counseling families and, in some situations, for modifying treatments.

## Author Contributions

All authors were involved in the writing, editing, and final approval of the manuscript.

## Conflict of Interest

The authors declare that the research was conducted in the absence of any commercial or financial relationships that could be construed as a potential conflict of interest.
